# Organosolv Fractionation of Birch Sawdust: Establishing a Lignin-First Biorefinery

**DOI:** 10.3390/molecules26216754

**Published:** 2021-11-08

**Authors:** Maxwel Monção, Kateřina Hrůzová, Ulrika Rova, Leonidas Matsakas, Paul Christakopoulos

**Affiliations:** Biochemical Process Engineering, Division of Chemical Engineering, Department of Civil, Environmental and Natural Resources Engineering, Luleå University of Technology, SE-971 87 Luleå, Sweden; maxwel.moncao@ltu.se (M.M.); katerina.hruzova@ltu.se (K.H.); ulrika.rova@ltu.se (U.R.); paul.christakopoulos@ltu.se (P.C.)

**Keywords:** organosolv pretreatment, birch, sawdust, fractionation

## Abstract

The use of residual biomass for bioconversions makes it possible to decrease the output of fossil-based chemicals and pursue a greener economy. While the use of lignocellulosic material as sustainable feedstock has been tried at pilot scale, industrial production is not yet economically feasible, requiring further technology and feedstock optimization. The aim of this study was to examine the feasibility of replacing woodchips with residual sawdust in biorefinery applications. Woodchips can be used in value-added processes such as paper pulp production, whereas sawdust is currently used mainly for combustion. The main advantages of sawdust are its large supply and a particle size sufficiently small for the pretreatment process. Whereas, the main challenge is the higher complexity of the lignocellulosic biomass, as it can contain small amounts of bark and cambium. Here, we studied the fractionation of birch sawdust by organosolv pretreatment at two different temperatures and for two different durations. We evaluated the efficiency of fractionation into the three main fractions: lignin, cellulose, and hemicellulose. The cellulose content in pretreated biomass was as high as 69.2%, which was nearly double the amount in untreated biomass. The obtained lignin was of high purity, with a maximum 4.5% of contaminating sugars. Subsequent evaluation of the susceptibility of pretreated solids to enzymatic saccharification revealed glucose yields ranging from 75% to 90% after 48 h but reaching 100.0% under the best conditions. In summary, birch sawdust can be successfully utilized as a feedstock for organosolv fractionation and replace woodchips to simplify and lower the costs of biorefinery processes.

## 1. Introduction

The search for renewable alternatives to fossil fuel-based resources has become an imperative in reducing greenhouse gas emissions and ensuring a sustainable future. In this respect, lignocellulosic biomass offers untapped possibilities as it is the most abundant crude feedstock and has been used successfully for the production of added-value chemicals, biofuels, and bioactive compounds [1,2].

According to the Food and Agriculture Organization, silver birch and downy birch account for 15.26% to 16.64% of native growing stocks in Finland, Sweden, and Norway [3]. In Sweden alone, the gross felling for the year 2020 was 93.3 million m^3^ and about 50% of that was processed in sawmills [4]. Around 8% of dry timber weight is turned into sawdust during processing at a typical sawmill [5], resulting in a significant amount of sawdust. Replacing woodchips with sawdust as feedstock for biorefinery applications has several advantages, and could dramatically improve the sustainability, economy, and efficiency of the overall process. First, sawdust is currently used mainly for combustion [5,6]. Second, by using sawdust, woodchips could be diverted to high-value applications, such as the production of paper or other cellulose-based items, where sawdust cannot be utilized. Third, woodchips must be milled to a smaller size before pretreatment, whereas sawdust particles are already suitably sized for pretreatment and fractionation, thus decreasing energy requirements and costs. Finally, the woodchips are made from the stem wood, whereas the sawdust is not as clean and can be a bit more complex since its composition may contain other parts of the tree such as small amounts of bark and cambium [7,8].

Pretreatment is a key process as it alters the compact lignocellulosic structure and facilitates access to its primary components: cellulose, lignin, and hemicellulose. Several physical, physicochemical, chemical, and biological pretreatment methods exist and can be combined for further optimization [9]. Organosolv pretreatment uses organic solvents that are feasible to recover, making it suitable for biomass biorefinery [10]. The organic solvent (ethanol, methanol, aceton etc.) added to the water solution facilitates separation of hemicellulose sugars, lignin and cellulose. Lignin and hemicelluloses are then recovered from the organic solution, while cellulose remains as solid biomass. When the solvent is distilled and recovered for further use, the lignin fraction can be separated as a solid material, while hemicellulosic sugars remain dissolved in water [11,12]. This strategy provides an easy and efficient separation of all three main lignocellulose components, allowing each of them to be utilized in separate applications [13,14].

In the present study, birch sawdust was subjected to organosolv pretreatment for different times and under various temperature and solvent conditions with the objective of evaluating its potential as feedstock for biorefinery. The results were compared to those obtained under similar conditions with milled birch woodchips, [11,12]. We demonstrate that sawdust represents a suitable alternative to woodchips and could substantially enhance the sustainability and rational flow of wood biomass in industrial and biorefinery processes.

## 2. Results and Discussion

### 2.1. Composition of the Pretreated Biomass

In our previous studies, organosolv pretreatment and organosolv pretreatment with steam explosion were tried on silver birch woodchips [11,12], resulting in a very promising fractionation output. Here, we evaluated the use of organosolv for the fractionation of birch sawdust as an alternative feedstock for biomass biorefinery processes, using different pretreatment temperatures (180 and 200 °C), times (15 and 30 min), ethanol concentrations (50% and 60% *v*/*v*), and presence or not of an acid catalyst (1% H_2_SO_4_ *w*/*w_biomass_*).

Table 1 summarizes the composition of pretreated biomass obtained under the different treatment conditions. Overall, in the absence of an acid catalyst, pretreated biomass contained more cellulose at 200 °C (57.98–69.20% *w*/*w*) than at 180 °C (44.16–57.18% *w*/*w*). Similarly, lignin and hemicellulose solubilization reached 80.71% (B0B6a) and 79.31% (B0B5a), respectively, at 200 °C, but only 60.32% and 54.46% (B1A5a), respectively, at 180 °C. Biomass pretreated at 200 °C contained 8.22–12.62% lignin and 12.96–22.23% hemicellulose.

Next, samples pretreated with 50% ethanol solution displayed a 9.6–13.4% higher cellulose content compared to those pretreated with 60% ethanol under the same conditions. This can be explained by the lower concentration of ethanol being characterized by increased availability of hydrogen ions and water, which lowers the pH and promotes the acid hydrolysis of hemicellulose [15]. When pretreatment took place at 200 °C, the use of 50% ethanol increased hemicellulose solubilization by 18.4% (15 min) and 22.1% (30 min) compared to use of 60% ethanol. Instead, when organosolv pretreatment was carried out at 180 °C, the results were not so clear-cut, with hemicellulose solubilization augmenting by 3.6% after 15 min, but decreasing by 10.9% after 30 min when ethanol increased from 50% to 60%. This could be attributed to the milder treatment conditions, which did not allow such extensive hemicellulose solubilization as at 200 °C. Lignin removal was better at higher temperature and ethanol content if it lasted 30 min; whereas only high temperature was beneficial for a 15-min treatment. On the contrary, at 180 °C, 50% ethanol promoted more extensive delignification. The lignin-carbohydrate bonds are readily hydrolysed under acidic conditions promoted by the lower ethanol content [15]; hence, the shift observed at 200 °C after 30 min could be explained by the severity of the pretreatment [16]. Generally, a longer treatment time improved hemicellulose solubilization by 2.1–9.3%, except at 180 °C with 50% ethanol, whereby a decrease was observed. Finally, treatment time did not have any major impact on lignin solubilization, except at 200 °C and 60% ethanol, whereby an increase of 10.5% was observed following prolonged treatment (Table 1). These results indicate that organosolv pretreatment could successfully fractionate birch sawdust, leading to the recovery of pretreated solids with very high cellulose content (69.2% *w*/*w*) but low hemicellulose (12.96% *w*/*w*) and lignin (13.24% *w*/*w*), as in sample B0B5a (Table 1). Lignin solubilization could be improved (80.17%) under the same treatment conditions upon replacing the 50% ethanol solution with 60% ethanol (B0B6a).

Finally, we evaluated whether addition of an acidic catalyst could further improve lignin and hemicellulose solubilization, while increasing cellulose content of pretreated solids. Addition of 1% H_2_SO_4_ *w*/*w_biomass_* to the B0A6a treatment (200 °C, 15 min, 60% ethanol) improved significantly the removal of lignin (87.91%) and hemicellulose (98.33%), resulting in pretreated solids with only 8.55% lignin and 1.61% hemicellulose, as well as 64.81% cellulose content (compared to 57.98% without acid). However, the harsher, acidic conditions promoted the unwanted extensive solubilization of cellulose (up to 44.16%). The severity of the treatment (presence of acid, higher temperature) can affect the recovery of biomass [17] as observed here for lignin solubilization following acid pretreatment (87.91%) or longer pretreatment at higher temperature (80.71%).

Matsakas et al. (2018) studied the effect of hybrid organosolv pretreatment of birch woodchips. The milled sample (<1 mm) was pretreated in a hybrid organosolv:steam explosion reactor. Pretreatment was performed at 200 °C, over periods of 15 to 60 min, and with ethanol content of 50%, 60%, and 70% *v*/*v*. The composition of untreated biomass was 34.7% *w*/*w* cellulose, 31.2% *w*/*w* hemicellulose, and 18.7% *w*/*w* lignin. The woodchips from that study and the sawdust from the present study yielded a similar composition of pretreated solids when pretreatment was operated under comparable conditions. For example, treatment at 200 °C for 30 min with 50% *v*/*v* ethanol resulted in a cellulose and hemicellulose content of 65.9% and 15.1% with woodchips, and 69.20% and 12.96% with sawdust. Lignin content, instead, was lower for woodchips (6.7% *w*/*w*) than for sawdust (13.24% *w*/*w*) [11]. Nitsos et al. (2016) studied the effect of time, ethanol load, and acidic catalyst on birch woodchips (<4 mm). The untreated biomass was composed of 35.2% cellulose, 28.0% hemicellulose, and 26.1% lignin. Under the best conditions (60% ethanol, 1% H_2_SO_4_, and 60 min at 182 °C), the cellulose content increased to 63.8%, while that of hemicellulose and lignin decreased to 1.1% and 16.0%, respectively [12]. Wen et al. (2013) carried out the organosolv pretreatment of woodchips obtained from the birch *Betula alnoides*. Prior to pretreatment, the milled wood (0.7–0.35 mm) was dewaxed with a solution of toluene and ethanol. The composition of untreated birch wood was 35.0% cellulose, 29.9% hemicellulose, and 25.4% lignin, which changed to 59.5% cellulose, 18.4% hemicellulose, and 14.2% lignin after pretreatment at 200 °C for 2 h with 60% ethanol [18]. In the present study, biomass pretreated at 200 °C displayed better fractionation results, as indicated by higher cellulose content, but lower hemicellulose and lignin content. In addition, no extra steps were required to further increase the cellulose content.

Smit and Huijgen (2017) subjected birch woodchips (<2 mm) to organosolv pretreatment at 140 °C for 2 h using 50% acetone solution and H_2_SO_4_ as catalyst. The composition of untreated biomass was 37.3% cellulose, 22.5% hemicellulose, and 27.2% lignin. Pretreatment achieved a significant improvement, as indicated by elevated cellulose recovery (87%) and removal of hemicelluloses (92%) and lignin (86%) [19]. Brienza et al. (2021) carried out the organosolv pretreatment of birch sawdust at 200 °C for 3 h using a 50% *v*/*v* butanol water solution and dithionite supplementation. The composition of untreated birch sawdust was 36.6% cellulose, 21.8% hemicellulose-derived sugars (xylose and arabinose), and 22.0% lignin. After pretreatment, the cellulose content increased to 75.8%, while that of hemicellulose-derived sugars and lignin decreased to 8.9% and 14.1%, respectively [20]. Although the cellulose content was elevated, the amount of dithionite (1 g per 3 g_biomass_) was very high and had a negative impact on costs. Moreover, ethanol is preferred to butanol due to its low cost and ease of recovery [10].

Thus, ethanol organosolv pretreatment of sawdust has a significant potential for scaling up as no extra steps (e.g., milling or extraction) are required before pretreatment and the overall results are similar or better compared to the options mentioned above.

### 2.2. Lignin Purity and Characterization

The detailed characterization of lignin in each sample (Table 2) revealed that the lignin obtained after pretreatment at higher temperatures (B0 samples) was generally of a higher purity compared to that obtained at the lower temperature (B1 samples). All samples showed a negligible amount of ashes (0.08–0.72%) and cellulose (0.00–0.50%), as well as a low hemicellulose content (0.32–7.04%).

The weight average (M_W_) of lignin increased with increasing temperature, ethanol concentration, and duration of pretreatment (Table 2). A shift from 180 °C to 200 °C resulted in the Mw increasing from 1800 g/mol (B1A5a) to 4500 g/mol (B0A5a). A shift from 50% to 60% ethanol led to the Mw increasing from 11,300 g/mol (B0B5a) to 13,300 g/mol (B0B6a). Prolonging the pretreatment from 15 to 30 min led to the highest increase in the Mw, from 4500 g/mol (B1A6a) to 15,900 g/mol (B1B6a). The number-average (M_n_) values followed the same trend as M_W_ values. In a previous study, the M_W_ and M_n_ values of birch lignin collected after organosolv pretreatment at 200 °C for 2 h in 60% ethanol solution were 3140 g/mol and 1240 g/mol, respectively, with a polydispersity index (PDI) of 2.52 [18]. These values are lower than those obtained in the present study at 200 °C for 30 min in 60% ethanol (B0B6a), whereby the M_W_ was 13,300 g/mol and the M_n_ was 1700 g/mol.

The PDI denotes the correlation between M_W_ and M_n_, and indicates the dispersity of molecular weights. At 200 °C, the PDI ranged from 3.75 to 8.21; while at 180 °C, it ranged from 2.28 to 8.53. However, under the same ethanol concentration and duration of pretreatment, a change in temperature led to a significant difference in PDI, as indicated by the two lowest values: 3.75 (B0A5a) and 2.28 (B1A5a). Sevastyanova et al. (2014) showed that a higher PDI value was indicative of more fragments of low-molecular-weight lignin produced during severe pretreatments, which is in agreement with the results of this study [21].

### 2.3. Sugar Recovery in the Hemicellulose Liquid Fraction

**Figure 1:**
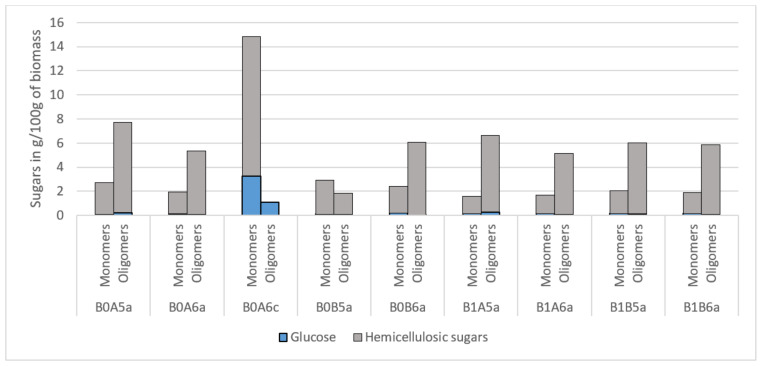
Total sugars (g/100 g of biomass; expressed as monomeric sugars) recovered after each pretreatment condition in the hemicellulose liquid fraction. Representation of the codes: 0—pretreatment at 200 °C; 1—pretreatment at 180 °C; A—pretreatment time of 15 min; B—pretreatment of 30 min; 5—50% *v*/*v* ethanol content; 6—60% *v*/*v* ethanol content; a—no catalyst added; c—1% *w*/*w_biomass_* H_2_SO_4_.

The amount of monomeric and oligomeric sugars present in the liquid filtrate after organosolv pretreatment was evaluated (Figure 1). The sample pretreated with acid catalyst (B0A6c) generated significant amounts of monomeric sugars, of which 11.54 g/100 g_biomass_ were from hemicellulose and 3.27 g/100 g_biomass_ were from cellulose. No oligomeric sugars of hemicellulosic origin were detected, whereas those derived from cellulose were only 1.07 g/100 g_biomass_. These values evidence the extent of biomass hydrolysis during pretreatment. In contrast, pretreatment without acid catalyst released roughly three times more oligomeric than monomeric sugars. This trend could be advantageous as it would allow the separation of oligomeric sugars and their subsequent utilization for prebiotic production [22]. In addition, non-acid pretreatment resulted in the release of mostly hemicellulosic sugars and only small amounts of glucose. This finding supports the results of biomass composition, which indicated a superior selective removal of hemicellulose, but greater cellulose retention in pretreated solids in the absence of acid catalyst. Overall, 0.17 to 4.34 g/100 g cellulosic sugars and 4.06 to 11.54 g/100 g hemicellulosic sugars were released during pretreatment. The recovery of cellulose and hemicellulose in the liquid fraction was as high as 0.54–12.21% and 18.0–40.5%, respectively, and could be further improved by increasing the temperature to 200 °C or by using 50% instead of 60% ethanol.

Nitsos et al. (2016) evaluated the filtrate liquor following organosolv pretreatment of differently sized milled birch (1 mm and 4 mm), at different ethanol contents, and in the presence or not of catalyst. Particle size, under the tested conditions, was shown to have very little effect on the amount of released hemicellulose- and cellulose-derived sugars [12]. In the absence of catalyst, hemicellulose-derived sugars were the main output, together with a very low amount of cellulose-derived sugars. The release of cellulose- and hemicellulose-derived sugars increased up to four and two times, respectively, when the catalyst was added. Finally, a higher amount of hemicellulose- and cellulose-derived sugars was released when the biomass was extracted with 50% as opposed to 60% ethanol solution [12]. Pretreatment was carried out at 182 °C for 60 min, which is long compared to the 15 and 30 min used in the present study. Cellulose-derived sugars released in the present study ranged from 0.06 g/100 g_biomass_ (60% ethanol) to 1.16 g/100 g_biomass_ (50% ethanol); whereas hemicellulose-derived sugars ranged from 1.06 g/100 g_biomass_ (60% ethanol) to 19.28 g/100 g_biomass_ (50% ethanol, 1% H_2_SO_4_).

Matsakas et al. (2018) evaluated the treatment of milled birch chips with hybrid organosolv:steam explosion and compared the effect of ethanol, time, and catalyst on the sugar concentration in the liquid fraction. Generally, very low amounts of cellulose-derived sugars, but significant amounts of hemicellulosic sugars, including up to six times more oligomeric sugars than monomeric sugars, were released during pretreatment. However, the addition of catalyst (0.2% *w*/*w_biomass_*) facilitated acidic hydrolysis of oligomers, reversing the monomeric-to-oligomeric sugars ratio from 0:8 to 10:1. Whereas Matsakas et al. (2018) observed five times more monomeric and two times more oligomeric hemicellulose sugars with increasing ethanol content; ethanol concentration did not exert a crucial influence on the concentration of released sugars in the present study, with hemicellulose sugars being released even at <50% ethanol [11].

### 2.4. The Overall Mass Balance

**Figure 2:**
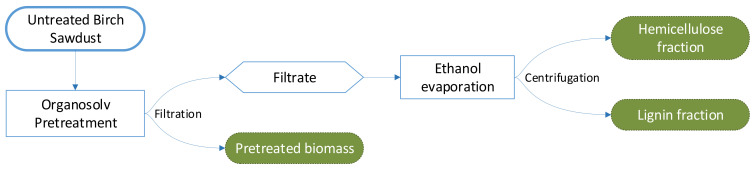
Diagram of the pretreatment process and obtained fractions.

Figure 2 summarizes the overall process and the three fractions (pretreated biomass, solid lignin fraction, and hemicellulose liquid) obtained from untreated birch sawdust biomass. Cellulose, hemicellulose, and lignin are the three main compounds. The organosolv pretreatment separates these compounds into distinct fractions. Accordingly, the pretreated biomass should consist mainly of cellulose, lignin should be recovered in the solid lignin fraction, and hemicellulosic sugars should dissolve during pretreatment and remain in the hemicellulose liquid [10].

**Figure 3:**
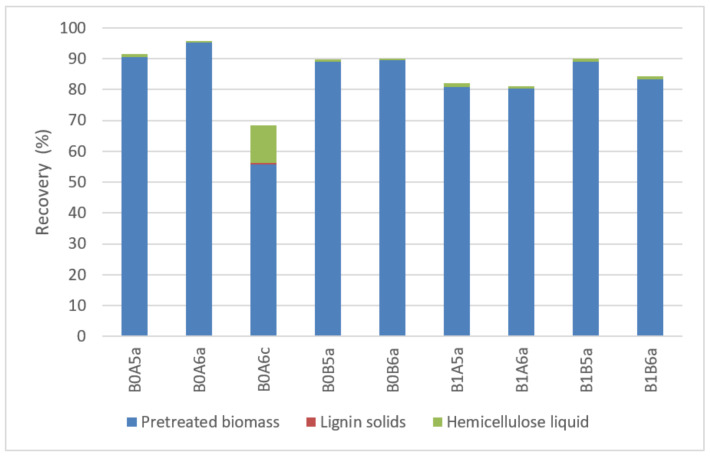
Cellulose distribution in the different fractions. Representation of the codes: 0—pretreatment at 200 °C; 1—pretreatment at 180 °C; A—pretreatment time of 15 min; B—pretreatment of 30 min; 5—50% *v*/*v* ethanol content; 6—60% *v*/*v* ethanol content; a—no catalyst added; c—1% *w*/*w_biomass_* H_2_SO_4_.

The recovery of cellulose was excellent, with most of it remaining in the pretreated biomass and only a residual amount being dissolved in the hemicellulose liquid, while trace amounts were detected in lignin solids (Figure 3). The use of acid during pretreatment lowered the total recovery of cellulose, as well as that from pretreated biomass. This can be attributed to the decomposition of cellulose to glucose (as evident also by the increased recovery of cellulosic sugars in the hemicellulose stream) and further breakdown of glucose into degradation products, which is promoted by the acid catalyst [23]. As a result, 55.8% of the recovered cellulose was present in the pretreated biomass, while 12.2% was dissolved in the hemicellulose liquid and trace amounts (0.3%) were found in the lignin fraction. On the contrary, in the absence of acid catalyst, more than 80% of cellulose was retained in the pretreated biomass fraction. The lowest cellulose recovery in the pretreated biomass (80.3%) was from samples pretreated at 180 °C for 15 min (B1A6a). Pretreatment at 180 °C resulted in biomass containing 80.3–89.1% cellulose; whereas pretreatment at 195–200 °C led to higher cellulose content (89.0–95.3%). The highest value (95.3%) was obtained following pretreatment at 200 °C, for 15 min, and with 60% ethanol solution (B0A6a). A minor amount of initial cellulose was recovered in the hemicellulose liquid (0.5–1.2%) and trace amounts were found in lignin solids (up to 0.3%). Overall, the decomposition of cellulose was significantly less pronounced in the absence of acidic catalyst.

**Figure 4:**
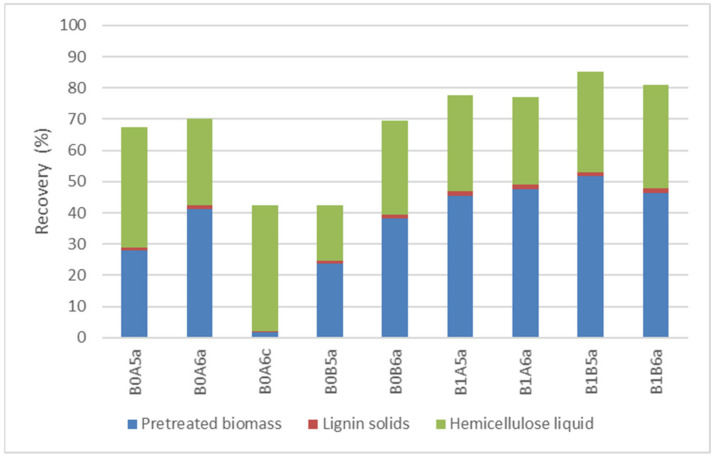
Hemicellulose distribution in the different fractions. Representation of the codes: 0—pretreatment at 200 °C; 1—pretreatment at 180 °C; A—pretreatment time of 15 min; B—pretreatment of 30 min;5—50% *v*/*v* ethanol content; 6—60% *v*/*v* ethanol content; a—no catalyst added; c—1% *w*/*w_biomass_* H_2_SO_4_.

Figure 4 presents the distribution of hemicellulose in the different fractions. The majority of hemicellulose (23.6–51.6%) was recovered in the pretreated biomass. A significant portion of the initial hemicellulose was disolved in the hemicellulose liquid (18.0–40.5%) and small amounts were recovered from lignin solids (0.8–1.6%). The use of acidic catalyst resulted in almost total removal of hemicellulose from the pretreated solids (1.7% recovery) and most of the remaining hemicellulose (40.5%) was recovered in the hemicellulose liquid. The lower pretreatment temperature led to a higher recovery of hemicellulosic sugars compared to the higher temperature. Given that hemicellulose is more susceptible to decomposition at higher temperatures and in the presence of acidic catalysts, its sugars (xylose, mannose, and arabinose) decompose two to four times faster than glucose at higher temperatures [23]. Therefore, 14.8–57.7% of the initial hemicellulose was lost during organosolv pretreatmet. In comparison, only 4.1–18.9% of the initial cellulose was lost to decomposition (wihtout catalyst).

**Figure 5:**
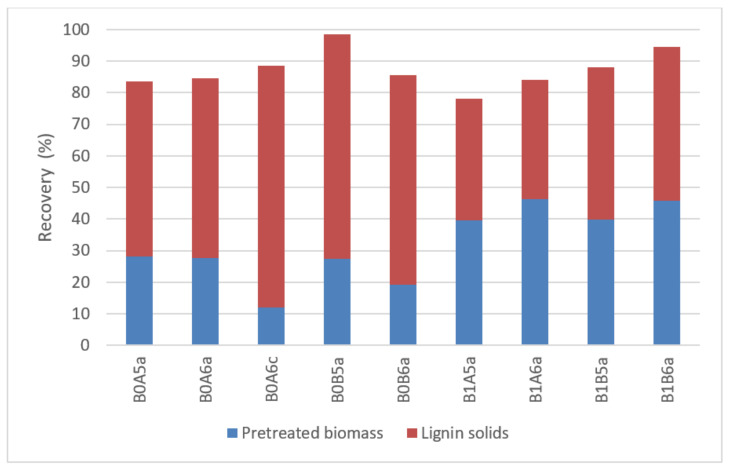
Lignin distribution in the different fractions. Representation of the codes: 0—pretreatment at 200 °C; 1—pretreatment at 180 °C; A—pretreatment time of 15 min; B—pretreatment of 30 min; 5—50% *v*/*v* ethanol content; 6—60% *v*/*v* ethanol content; a—no catalyst added; c—1% *w*/*w_biomass_* H_2_SO_4_.

Lignin distribution was examined in the pretreated biomass and lignin solids (Figure 5). In lignin fractionation, the temperature of pretreatment was crucial. Although overall lignin recovery was similar in all cases, its distribution varied substantially. While the lower temperature led to an even distribution of lignin between pretreated biomass (39.7–46.2%) and lignin solids (37.8–48.6%), the higher temperature shifted the majority of recovered lignin towards lignin solids (55.6–76.6%). A small amount of lignin (1.7–21.9%) was not recovered in either fraction and was dissolved in the hemicellulose liquid or decomposed during pretreatment.

The mass balance analysis indicates that a higher temperature during organosolv pretreatment leads to a better separation into the desired fractions. As a result, 90% of initial cellulose was recovered in pretreated biomass and 55.6–76.6% of initial lignin was recovered in lignin solids. However, as a downside, a higher temperature exacerbated the decomposition of hemicellulosic sugars. In comparison, a lower pretreatment temperature could successfully recover more than 80% of the initial cellulose, but the fractionation of lignin and hemicellulose was less efficient.

### 2.5. Enzymatic Sacchatification Efficiency

**Figure 6:**
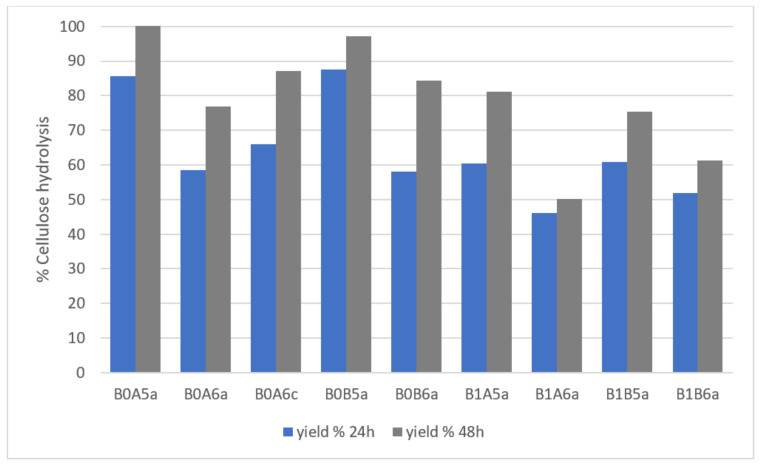
Enzymatic saccharification of the different pretreated biomasses. Representation of the codes: 0—pretreatment at 200 °C; 1—pretreatment at 180 °C; A—pretreatment time of 15 min; B—pretreatment of 30 min; 5—50% *v*/*v* ethanol content; 6—60% *v*/*v* ethanol content; a—no catalyst added; c—1% *w*/*w_biomass_* H_2_SO_4_.

Organosolv pretreatment yielded a birch biomass with high cellulose content, which is convenient for downstream bioconversion processes, such as ethanol fermentation or biogas production [11,24]. However, the efficiency of enzymatic saccharification used in such processes depends also on the lignin and hemicellulose content, as well as on pretreatment conditions. Therefore, as shown in Figure 6, the conversion of cellulose to glucose was evaluated for each sample after 24 and 48 h using an enzyme load of 20 FPU/g_biomass_ (Cellic^®^ CTec2). At both times, the yield was higher with biomass obtained following pretreatment with 50% rather than 60% ethanol solution. The highest yields, of 85.6% (24 h) and complete saccharification (48 h), were reached with biomass pretreated at 200 °C for 15 min in 50% ethanol solution (B0A5a), followed by a yield of 97.1% (48 h) achieved with the same treatment for 30 min (B0B5a). Generally, yields ranged around 75–90% after 48 h of enzymatic saccharification, with only samples pretreated at 180 °C and 60% ethanol achieving lower yields, such as 50.2% (B1A6a) and 61.4% (B1B6a). The use of an acidic catalyst improved the cellulose content, as well as the removal of lignin and hemicellulose sugars; the same trend was observed also during saccharification, with an increase of 12.6% at 24 h and 13.5% at 48 h compared to the same pretreatment without catalyst. These results suggest that the catalyst would cause more issues than advantages when scaling up the process, such as corrosion of the equipment and environmental pollution.

Raghavendran et al. (2018) studied the saccharification of birch chips milled at <1 or <4 mm and subjected to organosolv pretreatment at 182 °C for 60 min with 1% *w*/*w* H_2_SO_4_ as catalyst. After 48 h, yields of 95% and nearly 100% were reached with an enzyme load of 12.0 and 22.5 FPU/g_solids_ (Cellic^®^ CTec2), respectively [24]. Matsakas et al. evaluated the effect of hybrid organosolv:steam explosion pretreatment of birch chips milled to 1 mm and subjected to 200 °C with 60% ethanol for 15 min and 1% *w*/*w_biomass_* H_2_SO_4_ as catalyst. The final enzymatic hydrolysis was carried out with an enzyme load of 12.0 and 22.5 FPU/g_solids_ (Cellic^®^ CTec2), which resulted in yields of 97% and 100%, respectively [11].

Based on the above results, the yields obtained with birch sawdust are comparable to those obtained with milled chips. In addition, no catalyst is required during sawdust pretreatment to improve the enzymatic saccharification potential. Therefore, sawdust is well suited as a feedstock for bioconversions.

## 3. Materials and Methods

### 3.1. Feedstock

In the present work, residual sawdust of silver birch (*Betula pendula* L.) was used. The sawdust was collected from Swedish mills and originated from trees grown in southern Sweden. The sawdust was air-dried and then stored at room temperature. The composition of untreated birch sawdust was as follows (w/w): 37.07% cellulose, 30.87% hemicellulose, 22.6% lignin, and 1.09% ash, with an initial moisture of 8.26%. The size of sawdust particles was measured in a horizontal sieve shaker (Prüfsieb JEL 200; J. Engelsmann, Ludwigshafen, Germany), yielding the following distribution (in *w*/*w*): 0.7% of particles were >4 mm; 2.4% ranged from 2 to 4 mm; 76.7% ranged from 0.5 to 2 mm, and 20.2% were <0.5 mm.

### 3.2. Pretreatment

Sawdust was pretreated in an air-heated organosolv reactor within six 2.5 L metallic cylinders. Biomass dry weight was 110 g and was added in 1.1 L of either 50% or 60% *v*/*v* ethanol aqueous solution per cylinder (Table 3). The treatment was performed at 180 or 200 °C over 15 or 30 min in the absence of any catalyst. In one combination, the effect of an acidic catalyst (1% H_2_SO_4_ *w*/*w_biomass_*) was also studied. After pretreatment, the reactor was cooled to below 40 °C. Subsequently, the slurry was vacuum-filtered to separate the pretreated biomass from the liquor. The pretreated biomass was washed with 1.1 L of either 50% or 60% *v*/*v* ethanol aqueous solution, dried in an oven at 50 °C overnight, and stored in plastic bottles at room temperature. The ethanol was evaporated from the filtrate using a rotary evaporator (Heidolph, Schwabach, Germany) and lignin was recovered by centrifugation at 12,000× *g* for 10 min at 4 °C (5804R; Eppendorf, Hamburg, Germany). The solid lignin pellet was freeze-dried and stored at room temperature, while the hemicellulose liquid (containing the hemicellulose-derived sugars; both monomers and oligomers) were concentrated by water evaporation in a rotary evaporator and stored at 4 °C.

### 3.3. Enzymatic Saccharification Trials

The enzymatic saccharification potential of pretreated biomass was evaluated in 2-mL Eppendorf tubes containing 3% *w*/*w* biomass, 1.5 mL 50 mM citrate buffer (pH 5), and 20 FPU/g_solids_ of the commercial enzyme solution Cellic^®^ CTEc2 (Novozyme A/S, Bagsværd, Denmark). To prevent microbial growth, 0.02% *w*/*v* sodium azide was added to the solution. Incubation was performed in a thermomixer (Eppendorf) at 50 °C for 48 h. The samples were collected every 24 h and their sugar profile was analyzed by high-performance liquid chromatography (HPLC; PerkinElmer, Waltham, MA, USA) using an Aminex HPX-87N column (BioRad, Hercules, CA, USA) and refractive index detector (PerkinElmer series 200). The column was operated at 85 °C, with 0.1 M Na_2_HPO_4_ as the mobile phase, and a flow rate of 0.6 mL/min.

### 3.4. Analytical Methods

The untreated and pretreated biomass and lignin fractions were analyzed for cellulose, hemicellulose, lignin, ash, and moisture content using the NREL protocol [25]. The hemicellulose liquid was analyzed for monomeric and oligomeric sugars of both cellulosic and hemicellulosic origin. The sugar profile was analyzed by HPLC using an Aminex HPX-87H column and refractive index detector. The column was operated at 65 °C, with 0.005 M H_2_SO_4_ as the mobile phase, and a flow rate of 0.6 mL/min. Prior to HPLC analysis, the soluble oligosaccharides in the liquid were hydrolyzed with concentrated H_2_SO_4_ (4% *w*/*w*) at 121 °C for 1 h. When the sample was cooled down, it was neutralized with calcium carbonate, left to settle, and the supernatant was collected for analysis. The inorganic ash was determined gravimetrically after ashing at 550 °C for 3 h. The moisture content was determined gravimetrically after drying the samples at 80 °C overnight until constant weight was attained.

### 3.5. Gel Permeation Chromatography

Gel permeation chromatography was used to determine the molecular weight of lignin. First, the sample was derivatized by adding 0.9 mL glacial acetic acid and 0.1 mL acetyl bromide to 5 mg of lignin powder. The sample was stirred for 2 h at room temperature in closed vials. The solution was transferred to a round flask and evaporated in a rotary evaporator (Heidolph) at 50 °C and 50 mBar. Subsequently, the sample was washed twice with 1 mL tetrahydrofuran (THF) followed by solvent evaporation, dissolved in 1 mL THF, and filtered through 0.22-μm hydrophobic filters (Sartorius, Göttingen, Germany). Finally, the samples were analyzed by HPLC using a UV detector (set at 280 nm) and a Styragel^®^ HR 4E column (Waters, Milford, MA, USA), operated at 40 °C, with THF as mobile phase, and a flow rate of 0.6 mL/min. The calibration was done by using polystyrene (Sigma-Aldrich, St. Louis, MO, USA). The numbers were rounded up at 100 s due to the resolution of the method.

## 4. Conclusions

This study has shown that birch sawdust can be successfully utilized as a feedstock for organosolv fractionation in biomass biorefinery applications. Under the best conditions, the cellulose content was increased from 37.1% to 69.2% with the hemicellulose and lignin contents to be 12.96% and 13.24%, respectively. Subsequent enzymatic saccharification of the pretreated solids resulted in high glucose yields, ranging generally from 75% to 90% after 48 h, but reaching as much as 97.1% and 100% under the best conditions, which is ideal to be used as substrate for microbial cultivations. Moreover, fractionation after pretreatment yielded solid lignin of high purity with sugar impurities ranging from 0.82% to 7.22% and ash impurities from 0.08% to 0.72%, respectively. The separate hemicellulose liquid containing from 18.0% to 40.5% of hemicellulose and from 0.5% to 12.2% of cellulose origin sugars could be further utilized for downstream applications. Thus, in our work it was shown that sawdust can be used as a new feedstock for biorefineries after organosolv fractionation.

## Figures and Tables

**Table 1:** Biomass composition of solids following organosolv pretreatment.

Code	Biomass Solubilization (%)	Cellulose		Hemicellulose		Lignin		Ash (% *w*/*w*)	Total
		(% *w*/*w*)	Solub. (%)	(% *w*/*w*)	Solub. (%)	(% *w*/*w*)	Solub. (%)		
B0A5a	49.71	66.65	9.58	17.23	71.93	12.62	71.91	0.43	96.94
B0A6a	39.10	57.98	4.74	20.94	58.69	10.32	72.90	2.20	91.43
B0A6c	68.07	64.81	44.16	1.61	98.33	8.55	87.91	2.77	77.75
B0B5a	50.74	69.20	8.03	12.96	79.31	13.24	71.13	0.81	96.21
B0B6a	46.97	62.56	10.51	22.23	61.82	8.22	80.71	0.36	93.36
B1A5a	38.81	48.96	19.17	22.97	54.46	14.65	60.32	0.17	86.75
B1A6a	32.60	44.16	19.70	21.74	52.52	15.49	53.80	0.30	81.69
B1B5a	42.24	57.18	10.90	27.59	48.38	15.60	60.13	0.44	100.81
B1B6a	37.62	49.51	16.69	22.94	53.66	16.59	54.22	0.40	89.43
Sawdust	-	37.07	-	30.87	-	22.60	-	1.09	91.62

Representation of the codes: 0—pretreatment at 200 °C; 1—pretreatment at 180 °C; A—pretreatment time of 15 min; B—pretreatment of 30 min; 5—50% *v*/*v* ethanol content; 6—60% *v*/*v* ethanol content; a—no catalyst added; c—1% *w*/*w_biomass_* H_2_SO_4_.

**Table 2:** Composition of lignin obtained with each organosolv pretreatment. Weight average (M_W_), number-average (M_n_), and polydispersity index (PDI) of acid-insoluble lignin from birch sawdust were determined after acetobromination.

Code	Cellulose (%)	Hemicellulose (%)	Klason Lignin (%)	Ash (%)	M_W_ (g/mol)	M_n_ (g/mol)	PDI
B0A5a	0.08	1.85	95.18	0.47	4500	1200	3.75
B0A6a	0.14	2.32	83.86	0.27	8000	1100	7.34
B0A6c	0.50	0.32	86.44	0.42	9700	1400	6.97
B0B5a	0.39	1.56	92.00	0.72	11300	1400	8.21
B0B6a	0.00	2.01	91.90	0.31	13300	1700	8.04
B1A5a	0.14	3.55	77.93	0.12	1800	800	2.28
B1A6a	0.18	7.04	74.27	0.21	4500	1200	3.87
B1B5a	0.10	2.48	83.08	0.11	4600	1200	3.88
B1B6a	0.11	3.25	89.05	0.08	15900	1900	8.53

Representation of the codes: 0—pretreatment at 200 °C; 1—pretreatment at 180 °C; A—pretreatment time of 15 min; B—pretreatment of 30 min; 5—50% *v*/*v* ethanol content; 6—60% *v*/*v* ethanol content; a—no catalyst added; c—1% *w*/*w_biomass_* H_2_SO_4_.

**Table 3:** Experimental conditions during organosolv pretreatment.

CODE	Temperature (°C)	Time (min)	Ethanol Content (% *v*/*v*)	Catalyst (% *w*/*w_biomass_*)
B0A5a	200 °C	15	50	-
B0A6a	200 °C	15	60	-
B0A6c	200 °C	15	60	1% H_2_SO_4_
B0B5a	200 °C	30	50	-
B0B6a	200 °C	30	60	-
B1A5a	180 °C	15	50	-
B1A6a	180 °C	15	60	-
B1B5a	180 °C	30	50	-
B1B6a	180 °C	30	60	-

## Data Availability

Not applicable.

## Sample Availability

Samples of fractionated materials are available from the authors upon reasonable request.
